# Ovarian microcystic stromal tumor with significant bizarre nuclei: A case report

**DOI:** 10.1097/MD.0000000000021841

**Published:** 2020-08-21

**Authors:** Ying He, Lian Xu, Min Feng, Wei Wang

**Affiliations:** a Department of Pathology, West China Second Hospital of Sichuan University; b Key Laboratory of Birth Defects and Related Diseases of Women and Children (Sichuan University), Ministry of Education, Sichuan, China.

**Keywords:** immunohistochemistry, microcystic stromal tumor, ovarian tumor

## Abstract

**Rationale:**

Ovarian microcystic stromal tumor is a relatively rare tumor type, which is characterized by morphology with microcyst structure, solid cellular areas, and hyalinized fibrous stroma. The most reported tumors were stage I with good prognosis.

**Patient concerns:**

We report a case of a 33-year-old woman with primary ovarian microcystic stromal tumor with significant bizarre nuclei. We describe the clinical, histopathological, and immunohistochemical findings and review the English literatures. So far, as we know, the patient presented here is a rare case of ovarian microcystic stromal tumor with prominent bizarre nuclei accounting for about 50% of the tumor cells.

**Diagnoses:**

She was diagnosed with ovarian microcystic stromal tumor with significant bizarre nuclei.

**Interventions:**

The right ovarian tumor was resected laparoscopically on October 19, 2018.

**Outcomes:**

Up to now, the patient is free of disease at 19 months of follow-up.

**Lessons:**

This is a rare case of ovarian microcystic stromal tumor with obvious bizarre nuclei. This report will contribute to expand the morphological spectrum of ovarian microcystic stromal tumor.

## 1 Introduction

Ovarian microcystic stromal tumor (MCST) is a very rare subtype of ovarian pure stromal tumor, which was originally described in 2009 by Irving and Young^[1]^ and included in the category of ovarian stromal tumors of the 2014 World Health Organization Classification of Tumors of the Female Reproductive Organs. The histopathological morphology of this tumor varies with the relative prominence of 3 components: microcysts (dominant in 60% of cases), solid cellular regions, and hyalinized fibrous stroma.^[2]^ Based on personal experience of Young,^[3]^ the tumor cells usually have lightly eosinophilic cytoplasm and bland nuclei, but bizarre nuclei are present in 60% of the cases. Microcystic stromal tumors have a distinctive immunoprofile. They are typically negative for inhibin, calretinin, but diffuse positive for beta-catenin, cyclin D1, WT-1, FOXL2, and SF-1.^[4]^ Some pathologists think that this tumor may not be truly related to ovarian stromal origin but is, for now, placed in the stromal family as the “best fit.” ^[3]^ However, there is no specific description about the degree and area of bizarre nuclei in MCST in the English literatures. Here, we report a case of ovarian MCST with significant bizarre nuclei accounting for about 50% of the tumor cells.

## 2 Consent

The patient provided informed consent to collect data and images for publication. Ethical approval was not necessary in case of case report publication.

## 3 Case report

A 33-year-old woman was admitted to Guangyuan Traditional Chinese Medicine Hospital in October 2018 because of an abdomen mass. B ultrasound examination of the abdomen and pelvis confirmed a right ovarian mass. The detection of tumor markers (CA125, HE4, CA199, and AFP) were normal. She had been pregnant once and did not give birth to a child. Her previous medical history and family history were unremarkable. The ovarian tumor was then resected laparoscopically.

**Figure 1:**
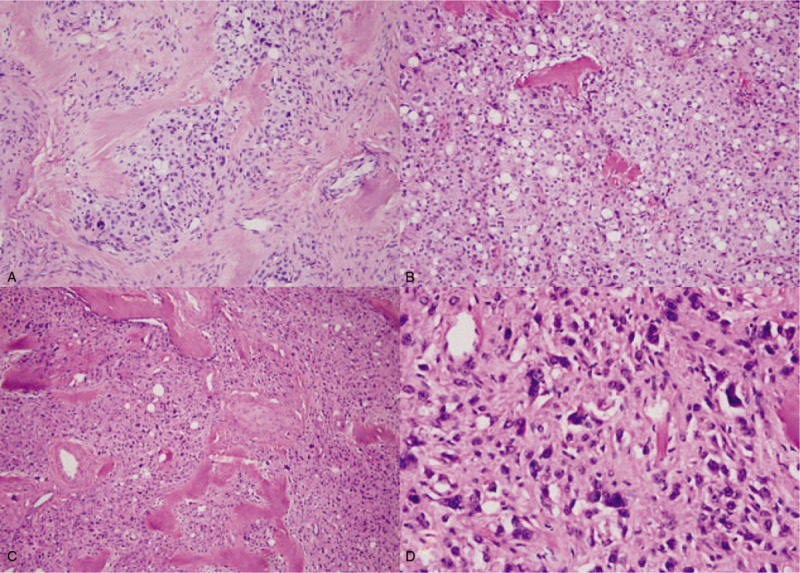
Ovarian microcystic stromal tumor. A, Microscopically, lobulated cellular regions separated by hyaline bands and fibrous plaques (H&E ×100). B, Typical microcysts structure (H&E ×100). C, Significantly degenerative bizarre cell areas (H&E ×100). D, The large pleomorphic “bizarre” nuclei unassociated with increased mitotic activity (H&E ×200).

Macroscopically, the tumor was well encapsulated, measured 3.2 × 3.0 × 2.6 cm. The cut surface of the mass revealed a tan-grayish appearance. Subsequently, the pathological HE sections of the patient were sent to the Department of Pathology in West China Second Hospital of Sichuan University for consultation. Microscopically, the tumor mainly consisted of 3 fundamental components: microcysts, solid cellular zones, and fibrous stroma. The nests and islands of cellular areas were intersected by collagenous stroma with hyaline plaques (Fig. 1A). Microcysts structure predominated and this pattern was characterized by small rounded to oval cystic spaces (Fig. 1B). Intracytoplasmic vacuoles were also frequently present like “signet-ring” cell appearance. About 50% of tumor cells contained lightly esosinophilic to pale cytoplasm with generally bland round to oval-shaped nuclei. However, obvious bizarre nuclei were present in about 50% of tumor cells (Fig. 1C and D). Mitosis was extremely rare (<1/50 high-power fields) in such areas.

**Figure 2:**
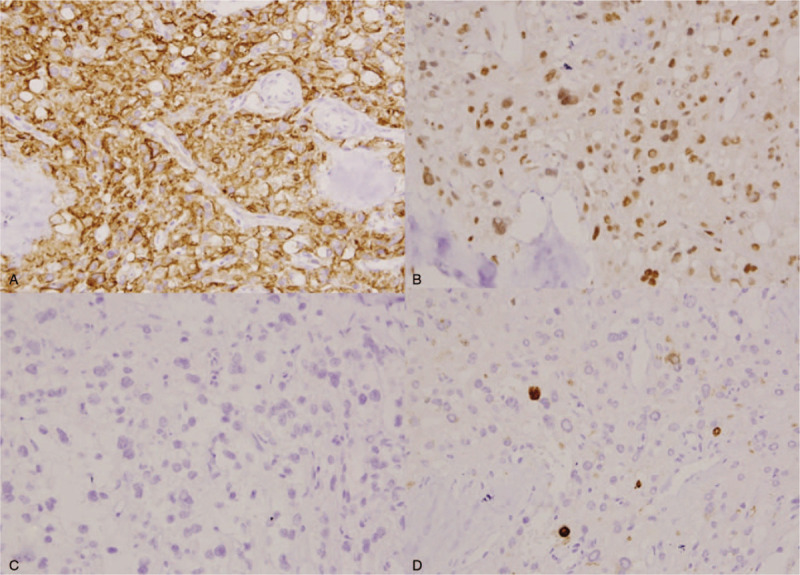
Immunophenotype of the ovarian microcystic stromal tumor. A, Positive staining for CD10 (×200). B, Nuclear positivity for WT-1 (×200). C, Negative staining for α-inhibin (×200). D, The proliferative index based on Ki-67 staining was about 2% (×200).

Immunohistochemically, the tumor cells were strongly positive for Vim, CD10 (Fig. 2A), WT-1 (Fig. 2B), CD56. They were negative for P-CK, EMA, α-inhibin (Fig. 2C), Calretinin, SMA, Des, S100, HMB45, ER, PR, SALL-4, CD117, PLAP, TFE-3. The proliferative index based on Ki-67 staining was about 2% (Fig. 2D). The histopathologic diagnosis was an ovarian microcystic stromal tumor. The patient was free of disease with a follow-up of 19 months.

## 4 Discussion

Microcystic stromal tumor is a very rare ovarian stromal neoplasm with less than 30 cases reported worldwide to date,^[5]^ which almost shows benign biological behavior. Although foci of bizarre cells were present in 60% of MCSTs,^[2]^ the case reported here contained significantly degenerative so-called bizarre cell areas accounting for about 50% of the tumor, in which there were large pleomorphic “bizarre” nuclei unassociated with increased mitotic activity. The mitotic rate of the tumor overall was typically minimal (<1/50 high-power fields). Apart from the presence of the prominent bizarre nuclei and multinucleated giant cells, the morphology and immunophenotype of the case were identical to classic ovarian MCST.

Sex cord-stromal tumors including granulosa cell tumors, Sertoli-Leydig cell tumors, and thecomas may exhibit focal degenerative bizarre nuclei.^[6,7]^ Young and Scully considered that the bizarre changes in these tumors resembled those seen in the uterine leiomyoma with bizarre nuclei, whose presence did not seem to adversely affect the prognosis of the underlying tumor.^[6,7]^ However, the degree of nuclear atypicality within granulosa cell tumors had been correlated with their prognosis in earlier English literature. Bjorkholm and Silfversward^[8]^ found that there was an 80% relative 25-year survival in cases with grade 1 nuclear atypicality in contrast to only a 60% survival in those with grade 2 atypia. But recent research suggested that only initial stage was found to be a significant prognostic factor of ovarian granulosa cell tumors according to multivariate analysis.^[9]^

In addition to classical histomorphological features comprising microcysts, solid cellular regions, and hyaline bands, McCluggage et al^[10]^ described 4 cases of MCST with variant morphology to expand the morphological spectrum of these tumors. The variants of MCST were characterized by diffuse, nested, corded, and tubular arrangements of bland epithelioid cells intersected by fibrous septa, with only minor cystic foci in 3 cases.^[10]^ Such neoplasms had the same immunophenotype of MCST with diffuse positive nuclear staining with beta-catenin, cyclin D1 and WT1, and diffuse staining with CD10; inhibin, calretinin were negative in all cases.^[10]^ At the same time, 3 of the cases exhibited CTNNB1 point mutations.

A heterozygous point mutation in exon 3 of the beta-catenin gene CTNNB1 had been identified in most MCSTs.^[4,11–13]^ This suggested that the Wnt/β-catenin pathway might play an important role in the pathogenesis of MCST. Meanwhile, a small number of cases of ovarian MCST had been reported in patients with familial adenomatous polyposis (FAP), an autosomal-dominant cancer predisposition syndrome caused by a germline mutation in the adenomatous polyposis coli (APC) gene on chromosome 5q21, illustrating that MCST might be an extracolonic manifestation of FAP.^[5,14,15]^ McCluggage et al^[13]^ found APC mutations occurred in a minority of MCST and were mutually exclusive with CTNNB1 mutation. Such results explained the nuclear staining with β-catenin in their all cases including those without CTNNB1 mutation, because either CTNNB1 or APC mutation could result in aberrant nuclear and cytoplasmic accumulation of β-catenin.

The MCST appeared to be clinically benign with an uneventful follow-up on the basis of limited experience. Exceptionally, Zhang et al^[5]^ reported a case of ovarian MCST with a recurrent intraovarian and extraovarian tumor after 9 years of follow-up, which suggested that MCST might have undetermined potential.

In summary, we presented a case of MCST with distinct morphology and immunophenotype, which contained significantly degenerative bizarre nuclei accounting for about 50% of the tumor cells. This report would expand the morphological spectrum of ovarian MCSTs.

## Author contributions

**Data curation:** Min Feng.

**Formal analysis:** Ying He.

**Funding acquisition:** Wei Wang.

**Supervision:** Wei Wang.

**Visualization:** Lian Xu.

**Writing – original draft:** Ying He.

**Writing – review & editing:** Lian Xu.
